# Mr. Wilkinson, on the Medicinal Powers of Electricity

**Published:** 1799-08

**Authors:** C. H. Wilkinson

**Affiliations:** No. 10, Leicester-street, Leicester-square


### *To the Editors of the Medical and Physical Journal.*

Gentlemen,

As I am extensively engaged in the medical application of electricity, if any observations I should be enabled to make should be deemed worthy of insertion in your Miscellany, I shall be happy to transmit to you, for your next, and every subsequent Number, the cases which occasionally come under my care.

I remain, Gentlemen,

Your most obedient,

*No.* 10, *Leicester-street, Leicester-square.* C. H. WILKINSON.

---

As my opinions of the influence of electricity on the human frame are, in some respects, different from those generally entertained, previous to entering into any investigation of its medicinal powers, I shall beg leave to premise a few general observations.

All fluids yet known, except air and oil, contain more or less electricity, and will freely admit its ingress, as well as egress. As the humau body is principally constituted of fluids, it is replete with electricity, and sensible of the least disturbance. A person insulated, giving a spark of electricity, communicates not identically the same portion he received from the machine, but an equal quantity, forced out of his body, by the impulse of that he received from the conductor. When thus connected with an electrical machine, a man becomes a part of the conductor—participates of the intensity—and equalizes with the whole.

Upon this consideration, we must regard the human body as a substance, throughout which electricity is diffused: such being the case, there can be no further addition, but an adequate portion will either be transmitted to some conductor, or form an electrical atmosphere round the body,

Obedient to the ſame general laws by which fluids are governed, the electric matter, upon any impulſe, moves in that direction where it meets with the leaſt reſiſtance; and, being an elaſtic fluid, the force of the impulſe will be in the inverſe cubic ratio of the diſtance of any part from the line of direction.

If a perſon takes a very gentle ſhock, he only experiences an uneaſy ſenſation at the tip of his fingers; if the ſhock is a little ſtronger, he feels it about his arms; if ſtronger, it agitates his body.

It is very eaſy to comprehend why we ſhould experience the electrical ſenſation at the extremities, when connected with the Leyden phial.— The quantity of electricity entering the body has in that part to overcome the reſiſtance of the electricity inherent in the fingers; from the fingers the impulſe is tranſmitted through the body: the fingers which are in connection with the negative ſide of the bottle, in paſſing out, have to overcome the reſiſtance of the egreſs.

In proportion as the impulſe is more violent, its effects will be more extended.

In the human body, we can either increaſe or diminiſh the natural quantity of electricity, or diſturb the relative ſituation of the whole.

The human body, like all conducting ſubſtances, is never found to poſſeſs in different parts, different ſtages of electricity, ſo as either, by a partial exceſs or diminution, to conſtitute a diſeaſe; hence the idea of equalizing the principle of electricity in the human frame, is unſupported by any logical experiment.

Electricity, unleſs from the impulſe of ſhocks, or the irritation of ſparks, never, either in a poſitive or negative ſtate, influences the pulſe; although Kratzenstein, Sauvages, Gerhard, and Cavallo aſſert the contrary. Their experiments were not very correctly conducted: it was accurately tried by the following gentlemen, viz. Drs. Deiman, Van Marum, Von Troopswyk, and Cuthbertson, with the powerful apparatus at Haarlem; the pulſe of no one was in the leaſt influenced either by negative or poſitive electricity. I have frequently tried myſelf, as well as others, in health or indiſpoſed, yet have never obſerved any increaſe in the circulation.

The effect of electricity is by diſturbing the natural quality inherent in any part of the human frame, and by thus altering the action of that part, inducing certain changes.

That

That ſuch changes may be conducive to health, it becomes requiſite for the adminiſtrator of medical electricity, to well aſcertain the ſeat of the complaint, and to know the different ſenſibilities of the different parts, and the effect of electricity upon them.

There are many complaints which would be conſiderably aggravated by the imprudent uſe of electricity, and a great number of other affections which could no ways be benefitted by this important agent, unleſs carefully applied.

If we were to apply electricity to the region of the diaphragm, in the ſame manner we would to a rheumatic affection of the extremities, what proſtration of ſtrength would be the conſequence! That exquiſitely ſenſible ſeptum, by ſuch an action, would be deranged in its functions, and reſpiration for a time impeded; it would not be again reſtored till the lungs were diſtended by a ſighing inſpiration, and the diſturbance ſoothed by a flood of tears.

So in paralytic affections, in any derangement of the nervous ſyſtem, to produce any good effect, the impulſe muſt be made on the ſource of the complaint. In the palſied extremity, to apply electricity to the foot alone, no advantage could ariſe; we ought in this, as in every other caſe, to attend to the ſource of the diſeaſe before we can afford the wiſhed-for relief.

Internal medicines are principally confined in their actions to the ſtomach—ſome few can be communicated to the lungs; to all other interior parts we poſſeſs no power of determining any particular medicines, unleſs electricity be regarded as ſuch. This principle we can direct in whatever manner we pleaſe. The muſcles, ligaments, or even ſolid bones are, as it were, capacious veſſels, affording eaſy tranſmiſſion to this fluid; and, as we can regulate its power at pleaſure, we are thus in poſſeſſion of an active, penetrating principle, by which we can produce a variety of actions in different parts.

It is a law in the animal economy, that two different actions cannot exiſt in any one part of the human frame at the ſame time; when the natural action is any ways altered, it will be removed by inducing another that will counteract it. We ought to be extremely careful that the action we induce be exactly proportionate to the nature of the derangement. If a part affected ſhould be in a ſtate of great irritability, or ſhould labour under any violent inflammatory action, theſe complaints would be aggravated by electricity. In all thoſe caſes which appear to be connected with diminiſhed powers of life, as in dull, deep-ſeated, obtuſe pains, or any interruption to the function of of the nervous system, or by the increase of any secretion, electricity is frequently beneficial.

Electricity must be regarded as a medicine whose properties are not as yet well ascertained, and whose effects on different constitutions not as yet determined;—such require the united observations of many individuals, before its influence on our organization can be properly known. On this account, those cases where it fails should be particularised, as well as those where it succeeds. Such is the plan I shall presume to adopt with whatever cases I may send for insertion to the Medical and Physical Journal.

CASE I.—*Hydrocele cured by Electricity.*

About eight months ago, a gentleman applied to me respecting an hydrocele, with a view of trying electricity. The testis was enlarged, and apprehended to be so diseased, that any operation for its radical cure was no-ways advisable. For two months I tried the effects of the electricity, without producing any other alteration than a diminution in the size of the testis; the dropsical accumulation appeared in some respects to be increased. He permitted me to puncture the scrotum with a small trochar. On the day after this operation, electricity was again had recourse to—a half-pint bottle, the electrometer at three-eighths of an inch. Shocks of this intensity, beginning at fifty, and gradually increasing to two hundred, were daily sent through the affected part: in the course of two months, the testis was reduced to the same size with the other. Electricity was suspended: no further tendency to accumulation has appeared.

N. B. Whenever the machine is not particularised, it is to be understood that a two-feet plate machine of Cuthbertson's was made use of; when any other size or form is employed, such will be particularised, as such very materially influences the intensity.

---

*To the Editors of the Medical and Physical Journal.*

GENTLEMEN,

